# Effects of vitamin E, inorganic selenium, bacterial organic selenium, and their combinations on immunity response in broiler chickens

**DOI:** 10.1186/s12917-018-1578-x

**Published:** 2018-08-24

**Authors:** A. M. Dalia, T. C. Loh, A. Q. Sazili, M. F. Jahromi, A. A. Samsudin

**Affiliations:** 1 Department of Animal Science, Faculty of Agriculture, Universiti Putra Malaysia, 43400 Serdang, Selangor Malaysia; 2 Department of Animal Nutrition, Faculty of Animal Production, University of Khartoum, Khartoum, Sudan; 3 Institute of Tropical Agriculture, Universiti Putra Malaysia, 43400 Serdang, Selangor Malaysia

**Keywords:** Bacteria, Broiler, Selenium, Vitamin E, Immunity, Cytokines

## Abstract

**Background:**

Selenium (Se) and vitamin E (Vit E) can act synergistically and affect biological processes, mainly antioxidant and immunity. The use of excess dietary Vit E and Se in animals’ feed could enhance immune response and induce disease resistance. Moreover, different Se sources may provide different alterations in the immune system. Accordingly, the aim of the current study was to assess the impact of dietary supplementation of Vit E, inorganic Se (sodium selenite, SS), bacterial organic Se of ADS18, and their different combinations on the plasma immunoglobulins, ceacum microbial population, and splenic cytokines gene expression in broiler chickens.

**Results:**

Present results showed that, Se and Vit E synergistic effect was clear in plasma IgM level at day 42 and in splenic cytokines expression (TNF-*α*, IFN-γ, IL-2, IL-10). The combination of 0.3 mg/kg ADS18-Se with 100 mg/kg Vit E showed the highest IgM level compared to Vit E- SS complex. The combination of either SS or ADS18-Se with Vit E had no significant effect on IFN- γ and IL-10 compared to Vit E alone, while Vit E alone showed the significantly lowest TNF-*α* compared to the Se combinations. Supplementation of 100 mg/kg Vit E had no effect on microbial population except a slight reduction in *Salmonella* spp. The main effect of Se sources was that both sources increased the day 42 IgA and IgG level compared to NS group. ADS18-Se modulate the caecum microbial population via enhancing beneficial bacteria and suppressing the *E-coli* and *Salmonella* spp. while both Se and Vit E factors had no effect on lymphoid organ weights.

**Conclusions:**

The inclusion of 100 mg/kg Vit E with 0.3 mg/kg ADS18-Se, effectively could support the immune system through regulation of some cytokines expression and immunoglobulin levels more than using ADS18-Se alone, while no difference was observed between using SS alone or combined with Vit E.

## Background

Infectious diseases are the leading causes of chick mortality. The immune system plays an important role in host defense against infectious agents. The immune system cells contain high polyunsaturated fatty acids in their cells membrane which increase their susceptibility to produce free radicals. Moreover, free radicals can be produced during the phagocytes action against the pathogens which lead to high oxidative stress [1]. It is well known that Se and Vit E can act synergistically and affect antioxidant and immunity response [2]. Vitamin E has been documented as a vital nutrient for the health, growth, and reproduction of all species of animals. This Vit is required to support the development of the nervous system and antioxidant system, as well contribute to disease resistance [3]. Selenium trace mineral plays an important role in antioxidant system through the selenoproteins, which are involved in redox regulation and antioxidant defense in all tissues and cells including the innate and adaptive immune cells [4]. The main antioxidant selenoprotein is glutathione peroxidase (GSH-Px), which reduce H_2_O_2_ and peroxides to the water and corresponding alcohols [5]. Antioxidant enzymes prevent reactive oxygen species production, which is formed normally during the body’s biological process [6]. Moreover, Se deficiency can damage the humoral immunity and cellular immunity, and deactivate B cells which results in immunoglobulin reduction [7].

The use of excess dietary Vit E and Se for enhancing the immunity and suppressing disease has been studied. Dietary Vit E and Se combination has been shown to improve the humoral immunity and provide better immunity response [8]. Vitamin E has been shown to increase antibody production by enhancing the humoral immune response or by acting as an adjuvant. Moreover, combined Vit E and Se significantly increased serum IgG and IgM levels in mice after their exposure to sodium azide which have an immune-suppressive effect [9]. In broiler chicken, higher antibody titer was observed when 0.06 mg/kg of Se was combined with 150 IU/kg Vit E in the diet, while higher cellular immunity was reported in the diet which included 300 IU/kg Vit E and 1 mg/kg Se [10]. Furthermore, the antibody titer increased when 125 mg/kg Vit E and 0.5 mg/kg Se were supplemented to the diet, and the author suggested that Se and Vit E combination improved the humoral immunity through the induction of immunoglobulins, especially IgM, which is released after the initial antigen exposure [11]. Selenium and Vit E together also may affect the proliferation of lymphoid cells and increase circulatory immunoglobulins and immune complexes, which would enhance antibody responses [8].

In the poultry industry, usually, Vit E is supplemented as α-tocopherol, which is the most abundant and bioavailable form, while dietary Se is supplemented as sodium selenite, however, recent studies demonstrated that organic Se such as seleno-aminoacids is more bioavailable than sodium selenite [12, 13]. Moreover, organic Se has the ability to accumulate Se in the animal’s tissues when the received amount of Se is higher than the body requirement [14]. Organic Se can be produced biologically through microbial reduction process. *Klebsiella pneumonia* (ADS18) isolated from hot spring water and was associated with high biomass organic Se-containing protein which can be used as Se source. Although it is well recognised that Se and Vit E interaction can enhance the antioxidant system, scientific data about the effect of Vit E and organic Se combination on broiler chicken is limited, and to the best knowledge of this researcher, no study has compared the combination of Vit E with organic Se versus inorganic source and no study use the bacterial organic Se in combination with Vit E. Accordingly, the aim of the current study was to assess the impact of dietary supplementation of Vit E, inorganic Se, bacterial organic Se of ADS18, and their different combinations on the plasma immunoglobulins, ceacum microbial population, and splenic cytokines gene expression in broiler chickens.

## Methods

### Birds and experimental design

Two hundred and sixteen (*n* = 216) day-old female broiler chicks (Cobb, 500) were sourced from a commercial hatchery (Ayamas LI Pt. Ltd.) and housed in battery cages. Each cage was equipped with one tube feeder and one bell drinker. On arrival, the chicks were individually wing banded, weighed, and randomly divided into six dietary groups, with each group having six replicates with six chicks per replicate. The experimental design was a factorial complete randomised design of two factors; Se (2 sources) x Vit E (2 levels). The birds had access to the feed and water ad libitum. All birds were subjected to vaccination against bronchitis (IB) and Newcastle disease (ND) on day 7, and against infectious bursal disease on day 14, through the intraocular route. A basal diet was formulated based on the nutrient requirements for starter and finisher stages (Table 1). The starter and finisher diets were offered to the broiler chickens from 0 to 21 and from 22 to 42 days of age, respectively. The treatment groups included T1 = basal diet, T2 = basal diet + 100 mg/kg α-tocopherol acetate, T3 = basal diet + 0.3 mg/Kg feed sodium selenite, T4 = basal diet + 0.3 mg /kg feed ADS18 Se, T5 = basal diet + 0.3 mg/kg feed sodium selenite+ 100 mg/kg α-tocopherol acetate, T6 = 0.3 mg/kg feed ADS18 Se + 100 mg/kg α-tocopherol acetate. The organic Se of ADS18 was prepared according to the method described by Dalia et al. [15], and administered orally equivalent to the dietary 0.3 mg/kg feed, based on the feed intake of the previous day. Other groups was treated orally using distilled water. The body weight was weighed individually and daily feed intake was recorded for each replicate.

**Table 1:** The ingredients of the basal diet

Ingredients	Starter %	Finisher %
Corn	52.5	56.250
Palm oil (Refine)	5.00	6.00
Soybean meal (44% cp)	32.50	30.00
Fish meal (58% cp)	5.15	3.25
L-Lysine	0.25	0.25
DL-Methionine	0.25	0.25
Dicalcium phosphate 18%	1.60	1.85
Calcium carbonate	0.60	0.35
Salt (Nacl)	0.30	0.30
Mineral Premix^a^	0.15	0.15
Vitamin Premix^b^	0.10	0.10
Toxin Binder^c^	0.15	0.15
Choline Chloride	0.10	0.10
Wheat pollard	0.135	1.00
Calculated nutrient content (g/kg DM)		
ME (kcal/Kg)	3081.1	3152.8
Crude protein	22.04	20.09
Crude fat	7.57	8.004
Calcium	1.189	1.0440
Phosphorus	0.786	0.768
Avail. P for Poultry	0.472	0.450
Analyzed Se (mg/kg)^d^	0.085	0.099
Analyzed vitamin E (mg/kg)^e^	224.9	167.1

^a^Mineral premix provided the following per kg diet: iron 120 mg, manganese 150 mg, copper 15 mg, zinc 120 mg, iodine 1.5 mg, and cobalt 0.4 mg

^b^Vitamin premix provided the following per kg diet: Vitamin A (retinyl acetate) 10.32 mg, cholecalciferol 0.250 mg, vitamin E (DL-tocopheryl acetate) 90 mg, vitamin K 6 mg, cobalamin 0.07 mg, thiamine 7 mg, riboflavin 22 mg, folic acid 3 mg, biotin 0.04 mg, pantothenic acid 35 mg, niacin 120 mg and pyridoxine 12 mg

^c^Toxin binder contains natural hydrated sodium calcium aluminium silicates to reduce the exposure of feed to mycotoxins

^d^The Se content measured using ICP.MS.

^e^Vit E content measured using spectrophotometer

### Samples and data collection

At day 21 and 42, 12 representative birds (selected randomly as 2 birds/pen) from each treatment were slaughtered for sampling in accordance with the procedures outlined in MS1500:2009 (Department of Standards Malaysia, 2009) which allows animal to be slaughtered by severing the jugular vein, without being stunned, with a razor sharp knife. In this study the slaughter was performed by a certified and highly experienced technician with a sharp knife. In this study, sustained absence of corneal reflex and rhythmic breathing were strictly monitored and checked to ensure that each individual bird was dead prior to further processing and sampling. Samples of blood, spleen, thymus, bursa of Fabricius, and ceacum digesta were collected and kept at − 80 C° for further analysis. Upon chicken slaughtering, part of spleen organ was collected in RNA-later Stabilisation Reagent (Qiagen, Germany) and processed for storage at − 80 °C, according to the manufacturer’s instructions.

### Plasma immunoglobulin concentration

Blood samples were collected at days 21 and 42 in vacutainer tubes containing ethylene diamine tetra acetic acid (EDTA). The samples were centrifuged at 3000 rpm for 15 min at 4 °C, and the plasma was stored at − 80 °C until antibody analyses. The chicken immunoglobulin (IgA, IgG, and IgM) were established employing commercial kits (Chicken IgA ELISA, Immunology Consultants Laboratory, Inc. USA), (Chicken IgM ELISA, Immunology Consultants Laboratory, Inc., USA), and (CEA544Ga, Enzyme-linked Immunosorbent Assay Kit, Cloud-Clone Corp., USA), respectively, following the manufacturer’s instructions.

### Analysis of caecal bacteria by quantitative real-time PCR

Collection of the caecal digesta was done directly after slaughter and kept at − 20 °C before it was used for quantification of caecal microbial population. The populations of *Lactobacillus* spp*., Bifidobacteria* spp*., Enterococcus* spp*., Enterobacteria* spp*., E. coli,.* and *Salmonella* spp. were established following the procedure reported by Navidshad et al. [16]. The genomic DNA extraction from the caecal digesta was done by using QIAamp® DNA Stool Mini kits following the manufacturer‘s instructions. DNA concentration and purity were measured with a Nanodrop ND-1000 spectrophotometer. Standard curves created by amplifying a known amount of target bacteria DNA were formed using serial dilution of PCR products from pure cultures of the targeted bacteria. The qPCR reaction was carried out using master mix of maxima SYBR® Green qPCR Master Mixes, ROX solution (Cat. no. K0252, Thermo Scientific Fermentas, UK). The primer sequences and annealing temperature of the targeted caecal bacteria used in this study are shown in Table 2. Each reaction volume comprised 12.5 μL of SYBR Green Master Mix, 1 μL of 10 μM forward primer, 1 μL of 10 μM reverse primer, 2 μL of DNA samples and 8.5 μL of molecular H2O. The qPCR assay was carried out with the BioRad CFX96 real-time PCR system (BioRad, USA) by utilising optical grade plates as follows: the reaction conditions consisted of an initial denaturation at 94 °C for 5 min, then by 40 cycles of denaturation at 94 °C for 20 s, primer annealing at 55 °C total bacteria, 58 °C for *Lactobacilli* spp*.,* 60 °C for *Bifidobacteria* spp*.,* and 50 °C for *Salmonella* spp., *E. coli, Enterococcus* spp*.,* and *Enterobacteria* spp*.* for 30 s respectively, and extension at 72 °C for 20 s. To verify the specificity of amplification melting curve analysis was performed following the last cycle of each amplification.

**Table 2:** The primer sequences of caecal targeting total bacteria, *Lactobacillus, Bifidobacteria, Enterococcus, Enterobacteriaceae, E.coli,* and *Salmonella*

Microorganism	Primer	Size of amplified fragments (bp)	References
*Lactobacillus*	F-5′-CATCCAGTGCAAACCTAAGAG-3′ R-5′-GATCCGCTTGCCTTCGCA-3′	341	[42]
*Enterococcus genus*	F-5′-CCCTTATTGTTAGTTGCCATCATT-3′ R-5′-ACTCGTTGTACTTCCCATTGT-3′	144	[9]
*Bifidobacterium*	F-5′- GGGTGGTAATGCCGGATG-3′ R-5′- TAAGCCATGGACTTTCACACC-3′	440	[43]
*Escherichia coli*	F-5′-GTGTGATATCTACCCGCTTCGC-3′ R-5′-AGAACGCTTTGTGGTTAATCAGGA-3′	82	[42]
*Enterobacteriace*	F- 5′-CAT TGACGTTACCCGCAGAAGAAGC-3′ R-5′-CTCTACGAGACTCAAGCTTGC-3′	195	[42]
Total *Salmonella*	F-5’-TCGTCATTCCATTACCTACC-3′ R-5’-AAACGTTGAAAAACTGAGGA-3′	119	[44]

### RNA isolation and real-time RT-PCR for cytokines gene expression

For RNA extraction, 30 mg of tissues was extracted using the RNeasy® Mini kit (Cat. No. 74104, Qiagen, Hilden, Germany). The concentration of the extracted RNA was examined with NanoDrop ND-1000 UV-Vis Spectrophotometer (NanoDrop Technologies, Wilmington, DE, USA) at 260/280 nm absorbance. Then I μg of extracted RNA was reversely transcribed employing the Quantitect® reverse transcription kit as per manufacturer’s instructions.

Real-time PCR was carried out with the use of Bio-Rad CFX96 Touch (Bio- Rad Laboratories, Hercules, CA, USA), in 96-well optical reaction plates. The GAPDH was taken as the reference gene to normalise the targeted genes. Primers were designed according to Table 3. Real- time qPCR analyses were conducted using 25 μL PCR master mix containing 12.5 μL SYBR Green Master Mix, 1 μL forward primer, 1 μL reverse primer, 2 μL template cDNA and 8.5 μL RNase-free water. Target genes were amplified using the following thermo cycling programme: 95 °C for 10 min, 40 PCR cycles at 95 °C for 10s, 60 °C for 15 s. Efficiency of amplification was established for each primer pair by utilising the serial dilutions of cDNA. The cycle numbers at which amplified DNA samples were in excess of the computer-generated fluorescence threshold level were normalised and comparisons made to establish the relative gene expression. Higher cycle number values showed lower initial concentrations of cDNA, and hence lowered levels of mRNA expression. Each sample was run in duplicate. The quantification of PCR reactions for each primer pair was carried out by comparing the target gene with the housekeeping gene. The formulation below was used to compute the gene expression of the target gene according to the method described by Pfaffl [17]:

$$ Ratio=\frac{{\left({E}_{target}\right)}^{{\varDelta CT}_{target}\left( Control- Treatment\right)}}{{\left({E}_{reference}\right)}^{{\varDelta CT}_{reference}\left( Control- Treatment\right)}} $$

**Table 3:** Primers used for qRT-PCR

Gene	size (bp)	Sequence (5′_3′)	References
IL-2	144	F- GTGGCTAACTAATCTGCTGTCCA R- CCGTAGGGCTTACAGAAAGG	[45]
IL-10	172	F- TAACATCCAACTGCTCAGCTC R- TGATGACTGGTGCTGGTCTG	[45]
IFN- γ	214	F- GAGCCATCACCAAGAAGATGA R- TAGGTCCACCGTCAGCTACA	[45]
TNF-α	–	F- GCTGTTCTATGACCGCCCAGTT R- AACAACCAGCTATGCACCCCA	[46]
GAPDH	312	F- CTGGCAAAGTCCAAGTGGTG R- AGCACCACCCTTCAGATGAG	[45]

The result was presented as a fold change between treatment and control group.

### Organ collection

Twelve birds of each treatment were slaughtered on day 42. The lymphoid organs (thymus, spleen, and bursa of Fabricius) were collected and weighed individually.

### Statistical analysis

The present study followed a factorial completely randomized design (3 (Se factors) × 2 (Vit E levels)). All data obtained were subjected to the generalised linear model (GLM) procedure of SAS (SAS, 2005). Tukey HSD test was used to separate means at *p* < 0.05 significance level.

## Results

### Plasma immunoglobulin concentration

The effect of Se and Vit E and their combination on plasma immunoglobulins in broiler chickens is shown in Table 4. Vitamin E supplementation had no effect on plasma immunoglobulin levels except significant (*P* < 0.05) elevation which was observed at day21 IgM. On the other hand, at day21 bacterial organic Se of ADS18 showed the highest IgA level compared to SS and NS groups with no significant differences between SS and NS treatments. At day 42, both ADS18-Se and SS showed significant (*P* < 0.05) elevation in IgG level than NS group, while SS showed the highest level of IgA and IgM however the differences between SS and ADS18-Se was in-significant. The interaction between Vit E and Se was significant (*P* < 0.05) on day 42 IgM. Without Vit E supplementation there were no significant differences between SS and ADS18-Se, however both of them were higher significantly than the NS group and Vit E alone. The combination of 0.3 mg/kg ADS18-Se with 100 mg/kg Vit E showed significantly (*P* < 0.05) highest IgM level compared to Vit E alone and Vit E- SS complex.

**Table 4:** Effect of dietary selenium sources and Vit E levels on antibody response in broiler chickens

Treatments		DAY 21			DAY 42		
		IgA (g/L)	IgG (g/L)	IgM (g/L)	IgA (g/L)	IgG (g/L)	IgM (g/L)
Vit E mg/kg							
0		1.135	4.239	0.837^b^	1.599	7.148	0.967
100		1.169	4.234	1.003^a^	1.524	7.279	1.038
Se sources							
NS		1.053^b^	3.695	0.875	1.357^b^	6.556^b^	0.941
SS		1.101^b^	4.439	0.966	1.645^a^	7.505^a^	1.040
ADS18-Se		1.302^a^	4.577	0.917	1.482^ab^	7.577^a^	1.027
SEM		0.039	0.179	0.032	0.074	0.181	0.020
*p Values*							
Vit		0.618	0.987	0.004	0.547	0.682	0.018
Se		0.019	0.105	0.378	0.011	0.028	0.042
Se*Vit		0.354	0.364	0.134	0.174	0.201	0.032
Vit E 0	NS	0.987	3.353	0.723	1.387	6.078	0.938^b^
	SS	1.153	4.690	0.888	2.033	7.690	0.999^a^
	ADS18-Se	1.263	4.675	0.899	1.378	7.676	1.036^a^
Vit E 100	NS	1.118	4.035	1.028	1.328	7.036	0.943^b^
	SS	1.048	4.187	1.045	1.659	7.321	0.940^b^
	ADS18-Se	1.342	4.479	0.936	1.588	7.479	1.017^a^
SEM		0.039	0.179	0.031	0.073	0.180	0.020

^a-b^means with different superscripts within a column-subgroup are significantly different (*P* < 0.05)

^a-b^means with different superscripts within a column of significant interaction are significantly different (*P* < 0.05)

*NS* no Se supplement, *SS* sodium selenite, *ADS18-Se* bacterial organic Se

Experimental unit, (*n* = 12)

### Ceacum microbial population

The main effect of dietary Se sources and Vit E levels on ceacum microbial population of broiler chickens is shown in Table 5. The *Lactobacilli* spp. in the group of birds treated with ADS18-Se was significantly higher than the SS and NS treated groups. Moreover, ADS18-Se supplementation was associated with the highest *Bifidobacteria* spp., and the lowest *E.coli* and *Salmonella* spp. compared to SS and NS groups, however, the difference between ADS18-Se and SS group was insignificant.

**Table 5:** Main effect of dietary selenium sources and Vit E levels on ceacum microbial population of 42-day broiler chickens

Parameters	Se			Vit E		SEM	*p values*		
Log 10 copy no/g ceacum	NS	SS	ADS18-Se	0	100		Se	Vit	Se*Vit
*Lactobacilli* spp.	6.91^b^	6.98^b^	7.74^a^	7.03	7.33	0.099	0.001	0.699	0.060
*Bifidobacteria* spp.	7.01^b^	7.57^ab^	8.21^a^	7.40	7.65	0.163	0.029	0.489	0.145
*Enterobacteria* spp.	4.20	4.02	4.53	4.23	4.25	0.087	0.146	0.914	0.784
*Enterococcus* spp.	6.86	7.25	6.88	7.08	6.76	0.081	0.128	0.118	0.240
*E.coli*	4.56^a^	4.33^ab^	4.21^b^	4.44	4.34	0.049	0.012	0.315	0.787
*Salmonella* spp.	3.41^a^	3.04^ab^	2.80^b^	3.38^a^	2.84^b^	0.105	0.031	0.006	0.126

^a-b^Means with different superscripts within a row-subgroup are significantly different (*P* < 0.05). *NS* no Se supplement, *SS* sodium selenite, *ADS18-Se* bacterial organic Se. Experimental unit, (*n* = 12)

On the other hand, dietary supplementation of Vit E had no effect on ceacum microbial population except for moderate reduction observed in *Salmonella* spp. count, which was significantly (*P* < 0.05) different compared to un-supplemented diet.

### Splenic cytokines gene expression

The effect of Se and Vit E on spleen cytokines gene expression is shown in Table 6. Selenium × Vit E interactions were significant (*p* < 0.0001) for a pro-inflammatory cytokine (TNF-*α*, IFN-γ) and anti-inflammatory cytokine (IL-2, IL-10). Without Vit E supplementation, Dietary Se of ADS18 down-regulated the TNF-*α* and IFN- γ significantly compared to SS and NS groups, and up-regulated the IL-2 and IL-10 significantly compared to other treatments, however, on IL-10 the effect of SS was higher compared to ADS18-Se. On the other hand, combination of either SS or ADS18-Se with Vit E had no significant effect on IFN- γ and IL-10 compared to Vit E alone, while Vit E alone showed the significantly lowest TNF-*α* compared to the Se combinations. Moreover, both Se combinations up-regulated IL-2 significantly compared to Vit E alone, but the SS combination was significantly higher than ADS18-Se.

**Table 6:** Effects of dietary selenium sources and vitamin E levels on cytokines gene expression of 42-day broiler chickens

Items		TNF-훼	IFN-γ	IL-2	IL-10
Vit E mg/kg					
0		0.879	0.786	2.019	2.066
100		1.100	0.808	3.019	4.609
Se sources					
NS		0.854	0.911	1.524	2.829
SS		1.134	0.798	3.029	3.633
ADS18-Se		0.981	0.682	3.006	3.551
SEM		0.226	0.169	0.259	0.296
*P values*					
Vit		0.0048	0.6935	<.0001	<.0001
Se		0.0133	0.0117	<.0001	<.0006
Se*Vit		<.0001	<.0001	<.0001	<.0001
Vit E 0	NS	1.000^a^	1.000^a^	1.000^c^	1.000^c^
	SS	0.992^a^	0.875^b^	1.203^b^	2.796^b^
	ADS18-Se	0.644^b^	0.483^c^	2.756^a^	2.402^b^
Vit E 100	NS	0.708^b^	0.822^b^	1.048^c^	4.658^a^
	SS	0.975^a^	0.720^b^	3.655^a^	4.470^a^
	ADS18-Se	0.918^a^	0.882^b^	2.257^b^	4.699^a^
SEM		0.061	0.042	0.258	0.294

^a-b^Means with different superscripts within a row-subgroup are significantly different (*P* < 0.05)

^a-c^means with different superscripts within a column are significantly different (*P* < 0.05)

*NS* no Se supplement, *SS* sodium selenite, *ADS18-Se* bacterial organic Se. Experimental unit, (*n* = 12)

Furthermore, it is clear that the combination of both sources of Se with the Vit E showed no better regulation compared to using each source alone, except for significant up-regulation observed on IL-10 of both sources, and up-regulation of 1 L-2 observed on SS.

### Body weight, feed intake and lymphoid organ weights

The effects of vitamin E, Se sources, and their different combinations on the body weight, feed intake and lymphoid organ weights are shown in Table 7. The final body weight was improved significantly (*P* < 0.05) when the diet was supplemented with Vit E compared to un-supplemented diet, Moreover, both SS and ADS18-Se showed high final body weight compared to NS group. The ADFI showed that dietary Vit E lowered ADFI significantly compared to un-supplemented diet, Moreover Se supplementation in the form of SS or ADS18-Se showed significant reduction in ADFI compared to NS diet.

**Table 7:** Main effect means of dietary selenium sources and vitamin E levels on body weight, feed intake and lymphoid organ weight of 42-day broiler chickens

Items (g)	Se			Vit E		SEM	*P values*		
	NS	SS	ADS18-Se	0	100		Se	Vit	Se* Vit
Initial BW	41.9	42.1	43.3	43.2	42.5	0.28	0.325	0.135	0.543
Final BW	2747.7^b^	2858.3^a^	2900.8^a^	2799.7^b^	2871.4^a^	21.79	0.004	0.048	0.565
Average DFI	98.34^a^	92.67 ^b^	90.89^b^	99.71^a^	94.23^b^	1.21	≤.0001	0.001	0.061
Lymphoid organ weight (% of BW)									
Thymus	0.204	0.169	0.209	0.205	0.178	0.014	0.483	0.367	0.272
Bursa	0.064	0.059	0.057	0.053	0.067	0.006	0.873	0.218	0.152
Spleen	0.123	0.137	0.114	0.115	0.138	0.010	0.755	0.365	0.788

^a-b^ Means with different superscripts within a row-subgroup are significantly different (*P* < 0.05)

*NS* no Se supplement, *SS* sodium selenite, *ADS18-Se*, bacterial organic Se

*BW* body weight, *DFI* daily feed intake per pen (*n* = 6)

All dietary Se sources and Vit E supplementation had no significant effect on lymphoid organ weights. Moreover, there were no synergistic effects between Se and Vit E on lymphoid organ weights.

## Discussion

Immune system is a main factor that influences the animal’s health and performance. It is well known that Se and Vit E can act synergistically and affect biological processes mainly, antioxidant and immunity [3]. Dietary Vit E and Se combination improved the humoral immunity and provided better immunity response through their role on free radical elimination and oxidative stress stability of the immune cells [8] Generally, the results of this study indicated that there are synergistic effects between Vit E and Se on plasma immunoglobulin, which appeared just in plasma IgM level Table 4. Combination of Vit E with bacterial organic Se of ADS18 significantly increased plasma IgM concentration in broiler chickens compared with the chicks fed SS- Vit E complex or Vit E alone. This result was consistent with the finding that Vit E and Se combination improved the humoral immunity and provided better immunity response [8, 11], when a dietary vitamin E (0, 125 and 250 mg/kg), selenium (Se, 0, 0.5 and 1 mg/kg), and their different combinations on humoral immunity was examined by intravenous injection of 7% sheep red blood cell (SRBC) followed by evaluation of serum for antibody titers in primary and secondary responses. Vitamin E and Se showed interactive effects on antiSRBC titers. Dietary vitamin E and Se alone also resulted in improvement of primary and secondary antibody responses [11]. On the other hand, Supplementation of 200 mg vitamin E/kg and 0·2 mg selenium/kg resulted in a significantly higher antibody titres, that associated with an increased serum concentration of total immunoglobulins and circulatory immune complexes [8]. Moreover, combined Vit E and Se, significantly increased serum IgG and IgM levels in mice after their exposure to sodium azide [9] .

Dietary supplementation of Vit E in broiler chicken improved primary and secondary antibody responses after heat stress challenge [18]. According to Habibian et al. [11], supplementation of Vit E at both 125 and 250 mg/kg levels showed no effect on IgM level in broiler chickens. Furthermore, Vit E supplementation had no significant effect on immunoglobulin levels of IgA, IgM, and IgG in the serum of duck [19]. This partially agreed with this study’s finding that there was no significant effect observed in IgA and IgG levels due to Vit E supplementation, however IgM level was significantly increased at day 21, but at day 42 the effect was significantly interactive with Se factor.

On the other hand, Khan et al. [20], demonstrated that dietary Se (inorganic Se) could improve immunity and significantly enhance the synthesis of IgA and IgG in broilers. This was clearly observed in this study, that Se supplementation of both inorganic and bacterial organic sources improved the bird’s immunity via IgA, IgG, and IgM elevation at day 42, but at day 21 bacterial organic Se showed significantly highest IgG level compared to SS and NS groups Table 5. However, our study concern about the different Se sources mainly the bacterial organic Se as unusual source of Se. This could be explained by the role of Se in protection and thus activation of B-lymphocytes cells which is the source of immunoglobulin [21]. Moreover, Se could increase the interleukin 2 receptors on the surface of lymphocytes [22].

The regulation of gut microbiota can be achieved via dietary supplements which have the ability to stimulate the growth of beneficial bacteria, or selectively can suppress the activity and growth of pathogenic bacteria. Trace elements as a diet component may affect the diversity of intestinal microbiota [23]. A study conducted by Molan et al. [24], revealed that the addition of sodium selenite or sodium selenate alone in the concentration of (1, 2, 3, 4, and 5 μg/ml) for each source, or their combination with China green tea to MRS culture of *Lactobacillus rhamnosus* and *Bifidobacterium breve,* enhanced their growth significantly compared to the control MRS*.* Oral supplementation of Se- china tea extract significantly (*P* < 0.05) increased the count of both *Lactobacillus* spp. and *Bifidobacterium* spp. in rat’s ceacum compared to tea extract without Se [25]. Moreover, bacterial culture of *Lactobacillus casei rhamnosus* showed improved cell viability when Se and cadmium (Cd) were added to the culture compared to the culture contained in the Cd alone [26]. These studies supported the finding of the current study that Se supplementation enhances the population of ceacum *Lactobacillus* spp. and *Bifidobacterium* spp. compared to the basal diet, but the Se sourced from ADS18 showed better count than SS treatment.

Furthermore, the current study showed that Se supplementation of inorganic and bacterial organic Se reduced the ceacum numbers of *E-coli* and *Salmonella* spp., but the difference was significant just between ADS18-Se and NS groups. In the same manner, feeding of probiotics or Se-enriched probiotics to piglets was associated with higher *Lactobacillus* spp. count and lower *E-coli* compared to the basal diet and sodium selenite diet [27]. Selenium-enriched probiotics also decreased *E-coli* via undefined antimicrobial metabolites [28]. In contrast, dietary Se-yeast in chickens showed no effect on the caecal microbiota or *Campylobacter jejuni* colonisation [29]. Intestinal microbiota, are probably sensitive to some trace elements such as Se, which is important for normal function for some bacteria, however, at the same time is considered a toxic element to other bacteria. Therefore, changes in dietary Se which acts as an antioxidant may modulate the diversity of intestinal microbiota via oxidative stress suppression and providing a better medium for the growth of beneficial bacteria. Lactic acids and *Bifidobacterium* spp. are able to incorporate Se from the growth medium to their cells [26, 30], which may enhance their growth and activity. Moreover, lactic acid bacteria have a role in inhibiting the colonization of pathogenic microorganisms through their secreted hydrogen peroxide, acids, and other antimicrobial substances. Published data on the effect of Vit E on caecum microorganisms are scant, thus, the reduction in *Salmonella* spp. count in the present study due to Vit E supplementation remain unclear.

Cytokines are small protein messengers released by the host as immune responses to infection, inflammation, and trauma. Some cytokines are anti-inflammatory and others are pro-inflammatory, and this also plays a role in the up-regulation of inflammatory reactions [31]. TNF-α, IFN-γ, and IL2 are pro-inflammatory cytokines that are produced by activated monocytes, macrophages, T cells, and natural killer cells. Adequate Se supplementation may enhance the immunity and decrease the host susceptibility to diseases [32]. Infected Se- deficient mice were associated with significant increases in the expression of cytokines IL-6, TNF-α, IFN-γ IL-18, and IL-10 in the liver, while Se adequate mice showed over-expression in the liver IL-10 [33]. However, in the present study, there was significant interaction between Se and Vit E on all examined splenic anti-inflammatory and pro-inflammatory cytokines expression, dietary Se of both ADS18 and SS down-regulated the IFN-γ and up-regulated IL-2 and IL-10 significantly compared to NS group, while ADS18 showed significant down-regulation of TNF-α level compared to both SS and NS groups. The down-regulation of pro-inflammatory cytokines observed in this study was due to IL-10 elevation, which is a known potent anti-inflammatory cytokine and deactivator of pro-inflammatory cytokine synthesis, macrophage, and monocyte [31]. However, the IL-2 up-regulation in this study was unexpected but according to Yang et al. [34], IL-2, is also required for the growth, proliferation, and differentiation of T cells, and the spleen sight is highly enriched with T cells, B cells, and monocytes.

On the other hand, Vit E supplementation down-regulated the pro-inflammatory cytokines expression in chickens receiving lipopolysaccharide [35]. The present study indicated that combination of either SS or ADS18-Se with Vit E had no significant effect on IFN- γ and IL-10 compared to Vit E alone, while Vit E alone showed the significantly lowest TNF-αcompared to both Se combinations. To the best of the researcher‘s knowledge, there is no report about the effect of Se and Vit E combination on splenic cytokines expression, however, previous studies indicated that dietary Vit E could lower pro-inflammatory cytokine expression through the NF-κB pathway alteration [36]. On the other hand, dietary Se-regulated inflammatory cytokines via NF-κB and MAPK signaling pathways [37]. Therefore, it is clear that, Se can induce immune response more than Vit E, through activation of anti-inflammatory cytokines and suppression of pro-inflammatory cytokines. The balance between anti-inflammatory and pro-inflammatory cytokines determines the severity of the disease.

In the current study, there was improvement in the final body weight and ADFI due to Vit E supplementation, and due to dietary Se either in bacterial organic or inorganic form. This was proved by the finding that supplementation of organic selenium and Vit E in the layer diet was efficient for improving performance [38]. Supplementation of 200 mg/kg of Vit E in broiler diet improved weight gain and feed intake compared to the basal diet [39]. In contrast, dietary Vit E and Se had no effect on the body weight of layer hens [40], and different Vit E levels showed no significant changes in the broiler performance [41]. On the other hand, present study showed that both Se and Vit E supplementation had no significant effect on the lymphoid organ weights (thymus, bursa, and spleen) in broiler chickens. These findings are consistent with previous results of [18] who revealed that Vit E supplementation could not affect the lymphoid organ. Moreover, Habibian et al. [11] indicated that dietary Se had no positive effect on the lymphoid organ weights under heat stress, however, the dietary inclusion of Vit E showed improvement of the relative weights of lymphoid organs. Along the same lines of the findings of this study, no synergestic effect between Se and Vit E were observed for relative lymphoid organ weights in the study conducted by Habibian et al. [11], however, Swain et al. [10] reported that dietary Se and Vit E had a synergestic effect on the lymphoid organ weights in broiler chickens under normal environmental conditions.The reason for this difference is not clear.

## Conclusion

In conclusion, the present study showed that dietary supplementation of bacterial organic Se of ADS18 in broiler chickens increased the ceacum beneficial bacteria, and support the immune system more than SS. The synergistic effect of Se and Vit E was apparent on the plasma IgM levels and splenic cytokines gene expression. The inclusion of 100 mg/kg Vit E with 0.3 mg/kg ADS18-Se, effectively could support the immune system through regulation of some cytokines expression and immunoglobulin levels more than using ADS18-Se alone, while there was no difference between using SS alone or combined with Vit E.

## Abbreviations

ADS18: Stenotrophomonas maltophilia
IFNγ: Interferon gamma
IgA: Immunoglobulin A
IgG: Immunoglobulin G
IgM: Immunoglobulin M
IL-10: Interleukin 10
IL-2: Interleukin-2
Se: Selenium
SS: Sodium selenite
TNFα: tumor necrosis factor alpha
Vit E: Vitamin E
